# Consumption of Enriched Yogurt with PAF Inhibitors from Olive Pomace Affects the Major Enzymes of PAF Metabolism: A Randomized, Double Blind, Three Arm Trial

**DOI:** 10.3390/biom11060801

**Published:** 2021-05-28

**Authors:** Maria Detopoulou, Agathi Ntzouvani, Filio Petsini, Labrini Gavriil, Elizabeth Fragopoulou, Smaragdi Antonopoulou

**Affiliations:** Department of Nutrition-Dietetics, School of Health Sciences and Education, Harokopio University, 17671 Athens, Greece; mdetopoulou@gmail.com (M.D.); agathintz@gmail.com (A.N.); petsini.filio@gmail.com (F.P.); labrinigbr@yahoo.gr (L.G.); efragop@hua.gr (E.F.)

**Keywords:** platelet-activating factor (PAF), PAF inhibitors, PAF enzymes, yogurt, olive pomace, LpPLA_2_, PAF-CPT

## Abstract

Platelet-activating factor (PAF), a proinflammatory lipid mediator, plays a crucial role in the formation of the atherosclerotic plaque. Therefore, the inhibition of endothelium inflammation by nutraceuticals, such as PAF inhibitors, is a promising alternative for preventing cardiovascular diseases. The aim of the present study was to evaluate the impact of a new functional yogurt enriched with PAF inhibitors of natural origin from olive oil by-products on PAF metabolism. Ninety-two apparently healthy, but mainly overweight volunteers (35–65 years) were randomly allocated into three groups by block-randomization. The activities of PAF’s biosynthetic and catabolic enzymes were measured, specifically two isoforms of acetyl-CoA:lyso-PAF acetyltransferase (LPCATs), cytidine 5′-diphospho-choline:1-alkyl-2-acetyl-sn-glycerol cholinephosphotransferase (PAF-CPT) and two isoforms of platelet activating factor acetylhydrolase in leucocytes (PAF-AH) and plasma (lipoprotein associated phospholipase-A_2_, LpPLA_2_). The intake of the enriched yogurt resulted in reduced PAF-CPT and LpPLA_2_ activities. No difference was observed in the activities of the two isoforms of lyso PAF-AT. In conclusion, intake of yogurt enriched in PAF inhibitors could favorably modulate PAF biosynthetic and catabolic pathways.

## 1. Introduction

Cardiovascular diseases (CVDs) are the main cause of death globally while early identification of individuals at risk of developing CVDs, and implementation of effective treatments is the prime objective of medical research [1]. In this context, behavioral risk factors such as unhealthy diet, physical inactivity, tobacco use and harmful use of alcohol could modify CVDs risk.

Platelet-activating factor (PAF), a phosphoglycerylether lipid (1-*O*-alkyl-2-acetyl-*sn*-glycero-3-phosphocholine) [2], constitutes a potent inflammatory mediator implicated in a plethora of pathophysiologic conditions, such as atherosclerosis [3], allergic disorders [4], cancer and other diseases. PAF is synthesized by almost all cell types, either at basal conditions or under specific stimuli. The main cellular sources of PAF are platelets, macrophages, monocytes, eosinophils, basophils, endothelial cells and renal cells. The levels of PAF in cells, tissues and biological fluids is regulated via its enzymatic biosynthesis by the remodeling and the de novo pathways and its catabolism [5].

In brief, initiation of the remodeling pathway leads phospholipase A_2_ to form lyso-PAF which is then acetylated by acetyl-CoA:lyso–platelet-activating factor acetyltransferases (Lyso-PAF AT) to generate PAF. Two isoforms of Lyso-PAF AT are known; one of them is activated under inflammatory conditions, whereas the other is calcium independent and is not implicated in inflammatory processes [5,6]. The crucial enzyme of the de novo pathway is cytidine 5′-diphosphocholine:1-alkyl-2-acetyl-*sn*-glycerol cholinephosphotransferase (platelet-activating factor–cholinephosphotransferase (PAF-CPT) which catalyzes the synthesis of PAF from 1-*O*-alkyl-2-acetyl-glycerol [7].

As far as PAF catabolism is concerned, an intracellular PAF-specific acetylhydrolase (PAF-AH) and its plasma isoform lipoprotein-associated phospholipase A_2_ (LpPLA_2_) are the main enzymes for the removal of the acetyl chain at the *sn*-2 position forming the inactive lyso-PAF [8]. In particular, the enzyme LpPLA_2_ is mainly associated with LDL, although it has also been associated with very low-density lipoproteins (VLDLs), intermediate-density lipoproteins, high-density lipoproteins (HDLs), and lipoprotein (a) [9,10]. Many studies have indicated that LpPLA_2_ is an independent predictor for coronary artery disease (CAD), with elevated LpPLA_2_ activity associated with an increased risk for CAD [11].

The role of diet in the development and progression of CVDs has long been studied [12,13], however, there is very limited evidence of the interactions between PAF and dietary compounds and their effect on CVD risk, especially in humans. 

Specific dietary compounds have been reported to modify PAF actions and/or metabolism in vitro and ex vivo [14,15,16,17,18]. Among them, it has been documented that polar lipid extracts from by-products of olive oil production exert in vitro inhibitory activity and antagonize PAF actions. Further analysis revealed that the inhibitory activity was attributed in specific polar lipids chemically characterized as glycerylether-*sn*-2-acetyl glycolipids [19]. In addition, the polar lipid extract of olive pomace that contains specific PAF receptor antagonists has been found to inhibit atherogenesis in hypercholesterolemic rabbits [20]. In human studies, consumption of Greek Mediterranean meals containing PAF inhibitors reduced platelet sensitivity against PAF in patients with type 2 diabetes mellitus and in healthy subjects [21,22]. Furthermore, the consumption of wines, with PAF inhibitors, along with a meal ameliorates postprandial platelet sensitivity against PAF [23] as well as the activities of Lyso-PAF AT and PAF-CPT in leucocytes of healthy men [24]. All the above support the idea that the consumption of Mediterranean foods that contain PAF inhibitors could have a protective effect on inflammation and thrombosis [16]. Especially, as far as PAF actions and metabolism are concerned, limited data exists [25,26]. 

Recent studies documented that production of yogurts from ovine milk using several starter cultures altered their composition in polar lipids resulting in higher inhibitory activity against PAF- and thrombin-induced human platelet-rich plasma aggregation [27,28]. Nowadays, bioactive polar lipids are added as functional ingredients in many foods, including dairy products [29]. For optimum health benefits, it is essential that the added bioactive lipids maintain their properties throughout food production and till consumption, as well as not adversely affect the desirable physicochemical and sensory attributes of the food product [30].

The aim of the present study is to evaluate the impact of a new functional enriched yogurt on PAF metabolism in humans. For this purpose, a randomized, three-arm, double-blind, placebo-controlled, parallel-group study was designed to evaluate if the daily consumption of a low-fat plain yogurt, enriched with the polar lipid extract of olive pomace could favorably modulate PAF biosynthetic and catabolic enzymes activity in middle-aged volunteers. 

## 2. Materials and Methods

### 2.1. Polar Lipid Extract of Olive Pomace

The polar lipid extract of olive pomace (OOPLE) used in yogurt enrichment was extracted from wet olive pomace (a semisolid olive oil by-product) of the Koroneiki variety from Crete (Greece) from olive mills that use a two phases centrifugal system for the delivery of oil using the modified counter current distribution method previously reported [31]. Briefly, olive pomace was initially extracted with absolute ethanol, filtered through a linen cloth and then by a filter press to remove solid residues, receiving the polar lipids extract in the filtrate. The filtrate was concentrated under vacuum to reduce the volume to approximately 1/3 and was then washed with n-hexane twice to remove the neutral lipid substances. The ethanol phase was concentrated under vacuum to remove ethanol and hexane residues, maltodextrin was added (30% *w*/*w*), the mixture was stirred sufficiently for homogenization and the OOPLE extract was stored at −80 °C until used. Carbohydrate content was determined according to the anthrone method and glucose was used for the standard curve [32]. Protein determination was based on Kjeldahl method 981.10 of the AOAC International. Fiber content was determined according to AOAC method 985.29. Total phenolic content was estimated using the modified method of Singleton and Rossi [33]. Ash and humidity determinations were based on methods 942.05 and 925.40 of the AOAC International, respectively. The method for the determination of fatty acid profile was based on ISO-12966 2. The amount of 100 g of the final OOPLE extract contains 56.9 g carbohydrates, 2.35 g proteins, 7.66 g polar lipids, 9.57 g dietary fibers, 6.7 mg phenolic compounds expressed as gallic acid, 17.2 g ash and 6.32 g humidity. Fatty acid analysis revealed that monounsaturated fatty acids were 71.8%, saturated FA 16.5% and polyunsaturated fatty acids 11.7%.

### 2.2. Production of the Enriched Yogurt

Low-fat plain strained yogurts, flavored with strawberry (strawberry fruit preparation), commercially available (Harmony 1%, MEVGAL SA, Koufalia, Greece) were used as placebo and also as a food matrix for the low-fat enriched yogurts (approximately 0.4% enrichment in OOPLE extract). Both yogurts, plain and enriched, which were provided during the whole duration of the intervention, were produced by a pilot scale manufacturing line and were physicochemically tested for their concentration in macronutrients and microbial parameters by the Greek dairy industry. The production of the enriched yogurt is fully described in the national patent titled “Method for the production of yogurt enriched with lipoid fractions derived from oil-press sub-products exhibiting antiatheromatic action” (Hellenic Industrial Property Organisation, OBI 1008550—25/08/2015; [34]). The fermentation procedure did not alter the biological activity of the OOPLE extract, tested as in vitro inhibition of PAF-induced aggregation in human platelet-rich plasma (PRP) and also the OOPLE extract did not affect the physicochemical and microbial parameters of yogurt [35]. The serving of both the plain and the enriched yogurt was 150 g and were isocaloric (85 kcal per serving). The final composition of the plain and enriched yogurt was identical, specifically, 100 g of both yogurts contain 1.0 g fat (saturated fat 0.6 g), 14 g carbohydrate (sugars 13 g), 5 g proteins and 0.1 g sodium. In addition, both yogurts contain 16% (*w*/*w*) strawberry supplement and also plain yogurt contained 2.1 ± 0.3 mg phenolic compounds/g of lyophilized sample while enriched yogurt contained 2.3 ± 0.2 mg phenolic compounds/g lyophilized sample. 

### 2.3. Participants and Settings

The study was carried out in accordance with the guidelines laid down in the Declaration of Helsinki (1989) of the World Medical Association, was approved by the Bioethics Committee of Harokopio University (40/30-10-2013) and was registered in ClinicalTrials.gov (NCT02259205). The study was conducted from October 2014 to June 2016 at the Department of Nutrition and Dietetics of Harokopio University in Athens, Greece. Volunteers were randomly allocated (block-randomization) into three intervention groups. The criteria of randomization were previously reported [36]. Participants in Group A (control) were advised to continue their regular diet and consume a maximum of one plain yogurt every 2 weeks. Participants in Group B (plain yogurt) were advised to continue their regular diet and consume one plain, non-enriched yogurt every day, and those in Group C (enriched yogurt) were advised to continue their regular diet and consume one enriched yogurt every day. The criteria for entering the study were previously stated along with the flowchart of volunteer recruitment [36]. Briefly, exclusion criteria were the history of cardiovascular or any other inflammatory disease, metabolic syndrome, presence of cold or flu, pregnancy, acute respiratory infection, dental problems, renal/hepatic diseases and medical treatment. The trial lasted 8 weeks. For safety monitoring reasons, all volunteers were questioned at 24 h telephone recalls for any potential side effects to the treatment, particularly gastrointestinal side effects. Additionally, gastrointestinal symptoms and possible side effects from yogurt consumption were assessed through validated questionnaires and stool frequency and consistency of evacuations using the Bristol Stool Scale were also recorded. No harms or unintentional side effects were reported by participants. Even though 92 volunteers were randomized into the three arms, specifically 31 in Group A, 30 in Group B and 31 in Group C, 4 of them (1 in Group A, 2 in Group B and 1 in Group C) were not included in the study because of lack of baseline data or because they did not complete the 8-week intervention. Volunteers’ adherence to the study protocol was assessed by two 24 h recalls every two weeks conducted by trained personnel. Blood samples were collected at baseline, at the 4th week and at 8th week of the intervention. 

### 2.4. Randomization and Blinding

The participants were randomly allocated to the intervention groups using stratified randomization scheme by age, sex, body mass index (BMI) and menstrual cycle among the three intervention groups. Specifically, a computer-generated sequence of random numbers from the Uniform (0,1) distribution was created using a specific software command runiform() and then, this sequence was transformed to discrete values 1, 2, 3 (through tertiles), representing each intervention Group.

The trial was double-blind. A researcher who was not involved in the biochemical and nutritional analyses was responsible for the enrollment, randomization of the participants, and for the data entry, whereas, all other investigators and participants were blinded to the allocation to intervention groups. Similarity of the interventions was achieved by using the same packing of yogurt into all groups.

### 2.5. Anthropometric Measurements and Biochemical Measurements

Anthropometric measurements, BMI calculation and classical biochemical parameters were evaluated as previously described [36]. LDL-cholesterol (LDL-C) was calculated using the Friedewald formula. 

### 2.6. Isolation of Serum from Whole Blood

For the isolation of serum, 10 mL of fasting venous blood were drawn into vacutainer with no anticoagulant, and serum was collected after 45 min incubation at room temperature by centrifugation at 1500× *g* for 10 min. Serum aliquots were stored at −80 °C until analysis.

### 2.7. Isolation of Leukocytes from Heparinized Blood

The procedure used has been already reported [25,37]. Briefly, 5 mL of heparinized blood were obtained from each volunteer, dextran solution was added to induce erythrocyte sedimentation and leukocytes were washed and isolated by subsequent centrifugations and homogenized with sonication. The leukocyte homogenates were aliquoted and stored at −80 °C. Protein concentration of all homogenates was determined according to the Bradford method with the use of bovine serum albumin as protein standard [38].

### 2.8. Measurement of Platelet-Activating Factor—Acetylhydrolase Activity in Leucocyte Homogenate

Determination of PAF-AH activity was performed as previously described [25] based on the trichloroacetic acid precipitation method using [^3^H] PAF as a substrate. All assays were performed in duplicate. The enzyme activity was expressed as specific activity: pmol of PAF degraded per mg of leukocyte homogenate protein per min (pmol/mg/min).

### 2.9. Measurement of Lipoprotein Associated Phospholipase A_2_ Activity in Serum

Serum LpPLA_2_ activity was measured by a commercial kit using 2-thio PAF as a substrate (Cayman Chemical, Michigan, MI, USA). The intra-assay CV was <4% and the interassay CV was <10%. All assays were performed in duplicate. The enzyme activity was expressed as specific activity nmol of PAF degraded per min per mL of serum (nmol/mL/min).

### 2.10. Assay of Lyso-Platelet-Activating Factor Acetyltransferase Activity in Leucocyte Homogenate

Two isoforms of Lyso-PAF AT were determined as previously described [25], one of them is activated under inflammatory conditions (Lyso-PAF ATC), while the other one is calcium independent (Lyso-PAF ATE). Briefly, isolated leucocyte homogenates, 15 μg of total protein, were incubated with lyso-PAF and acetyl-CoA. In the case of Lyso-PAF ATC, the assay was performed in the presence of 2.8 mM CaCl_2_, and in the case of Lyso-PAF ATE, the assay was performed in the presence of 1.4 mM EDTA. Cold chloroform:methanol (2% acetic acid) was added for stopping the reaction. All assays were performed in duplicate. The enzyme activity was expressed in pmol/mg/min.

### 2.11. Assay of Platelet-Activating Factor Cholinephosphotransferase Activity in Leucocyte Homogenate

Determination of PAF-CPT activity was performed as previously described [25]. Briefly, 15 μg of isolated leucocyte homogenate protein was incubated at 37 °C for 5 min in the presence of dithiothreitol, EDTA, cytidine 5′-diphospho-choline and 1-O-hexadecyl-2-acetyl-*sn*-glycerol in total reaction volume of 200 μL. Cold chloroform:methanol (2% acetic acid) was added for stopping the reaction. All assays were performed in duplicate. The enzyme activity was expressed in pmol/mg/min.

### 2.12. Extraction and Quantification of Platelet-Activating Factor

The extraction of the PAF produced during the enzymatic assays of Lyso-PAF ATs and PAF-CPT was based on the acid Bligh–Dyer method [39], and PAF was subsequently determined with liquid chromatography (LC)-MS as previously described [25]. Quantification was performed using the transition m/z 524.37 at retention peak time of 10 min. A calibration curve, with range 1.6–24 pmol PAF, was routinely performed every two batches and a control PAF sample was included in each batch. Inter-day and intra-day precision were 15.9 and 11.8%, respectively. Mass spectra were processed using the Xcalibur 4.0 (Thermo Scientific, Waltham, MA, USA) software. The enzymatic activity was expressed as specific activity pmol PAF/mg per min (pmol/mg/min).

### 2.13. Statistical Methods

All statistical analyses were performed using Stata Statistical Software, Release 12 (StataCorp LP: College Station, TX, USA). The significance (α) level was set to 0.05 for all two-tailed tests. The Shapiro–Wilk W test for normality as well as normal quantile plots were used to assess the normality of data. A priori statistical power analysis showed that 25 participants in each arm (i.e., 75 in total) were adequate to achieve statistical power equal to 83% at 5% significance level of two-sided hypotheses that evaluated 1 standard deviation (SD) differences based on EC_50_ values of platelet aggregation induced by PAF (primary outcome of the study) among groups.

Participants’ characteristics are described at baseline and after 4 and 8 weeks on each dietary intervention. Categorical variables, i.e., sex and intervention group, are presented as absolute frequencies (*n*); continuous variables are presented as mean (standard deviation) for normally distributed data or as median (25th, 75th percentiles) for data which deviate from the normal distribution. The Pearson’s chi-square test was used to assess independence between sex (2 levels) and intervention group (3 levels). The one-way analysis of variance (ANOVA) was used to determine whether there are any significant differences among the intervention groups on normally distributed variables. The Kruskal–Wallis H test was used to determine if there are any significant differences among the intervention groups on non-normally distributed variables.

The Skillings–Mack test was used to assess changes across baseline, week 4 and week 8 on each dietary intervention, and only significant changes are reported. Subsequently, Wilcoxon matched-pairs signed-ranks tests were performed to assess changes between week 4 and baseline, as well as between week 8 and baseline; results are reported as percent change (% change), calculated as the change between values at the 4th or 8th week of the dietary intervention and values at baseline, and are presented as median (25th, 75th percentiles).

Associations between enzyme variables evaluated at baseline and potential covariates, i.e., sex, age and BMI at baseline, were assessed. Wilcoxon rank-sum test was performed to compare ranked enzyme values at baseline between men (*n* = 46) and women (*n* = 42); results are reported as median (25th, 75th percentiles), and statistical significance is reported as the unadjusted *p*-value. Box plots were also created to visually inspect the distribution of baseline values for each enzyme between men and women. Spearman’s rank correlation was used to assess the association between each enzyme variable and age or BMI at baseline, as well as between enzyme activities at all time points in the three intervention groups. Scatterplots with linear predictions were also created to visually inspect these associations. Finally, correlations between the ranked values of the enzyme variables were evaluated for all participants at baseline, and by intervention group at week 4 and week 8 of the study; adjustments were made for sex, age and BMI.

Median regression analysis was performed in order to estimate the size of the difference between the intervention groups. First, boxplots were created for each of the enzyme variables evaluated at baseline, week 4 and week 8 of the study separately for each of the three intervention groups. The distribution of each enzyme variable for the “Control”, “Plain yogurt” and “Enriched yogurt” groups had similar shape. Subsequently, median regression was performed in order to estimate the difference in medians between the intervention groups, i.e., “Enriched yogurt versus Control”, “Plain yogurt versus Control”, “Enriched yogurt versus Plain yogurt”, for each enzyme variable measured at week 4 and week 8 of the study. Results are presented as difference in medians (95% confidence interval of the difference) between the intervention groups; a Bonferroni correction was applied to the observed *p*-value based on the number of regressions performed for each enzyme variable (i.e., 3 regressions for 3 pairs of groups). Sex and enzyme variable values at baseline were included in the regression models as covariates.

The analyses were carried out according to the intention-to-treat (ITT) approach [40]. Participants who were randomly assigned to any of the 3 study groups and had at least one measurement at baseline and at one of the planned time points were studied. Missing values were imputed using multiple imputations method, which involved fitting a statistical model to the observed data by means of a mixed effect model with repeated measures (MMRM) approach. The MMRM included fixed categorical effects of the 3 treatment groups, 2 time points and treatment-by-time point interaction, as well as the continuous fixed covariates of baseline values and baseline value-by-time point interaction.

## 3. Results

The flow diagram of the study is presented in Figure S1.

### 3.1. Characteristics of the Participants at Baseline, Week 4 and Week 8 of Intervention

There is no significant association between sex and intervention group, i.e., men and women are equally represented in each intervention group. There are no significant differences among the intervention groups with respect to age (in years), and LDL-C (in mg/dL) at baseline, week 4 and week 8 of the intervention (Table 1, Table 2 and Table 3). Significant differences were found for Lyso-PAF ATC at baseline and at week 4 (Table 1 and Table 2) and for LpPLA_2_-to-LDL ratio at week 8 (Table 3).

No significant changes were found for the activities of the enzymes at 4 and 8 weeks, compared to the baseline levels, in the enriched yogurt and the plain yogurt groups. In the control group, the specific activity of PAF-AH increased by 12.3% (25th, 75th: 0.2%, 31.3%; *p*_within-group_ = 0.004) during week 4 compared to baseline, whereas the LpPLA_2_-to-LDL ratio increased by 3.8% (25th, 75th: −3.2%, 12.5%; *p*_within-group_ = 0.011) during week 8 compared to baseline. 

### 3.2. Associations between Enzyme Variables and Covariates (Sex, Age, BMI) at Baseline

The activity of LpPLA_2_ and LpPLA_2_-to-LDL ratio were higher in men compared to women (*p* = 0.017 and *p* = 0.013, respectively). All the other metabolic enzymes of PAF did not show any significant relationships with either age or BMI measured at baseline (*p* > 0.10, for all bivariate correlations). These results were also graphically verified (Figures S2–S4).

### 3.3. Estimation of the Size of the Difference between the Intervention Groups

At week 4 of the study, the median PAF-CPT specific activity of the participants who received the enriched yogurt was lower than the corresponding one of the participants in the control group (*p* = 0.023) (Table 4) after adjustment for the baseline values of PAF-CPT. At week 8 of the study, the median PAF-CPT specific activity of the participants who consumed either the enriched yogurt or the plain yogurt was lower than the PAF-CPT activity in the control group (*p* = 0.043 and *p* = 0.048, respectively) after adjustment for the baseline values of PAF-CPT (Table 5). In addition, the intake of the enriched yogurt resulted in lower LpPLA_2_-to-LDL ratio at 8 weeks compared to the plain yogurt (*p* = 0.010) after adjustment for the corresponding values at baseline (Table 5).

### 3.4. Correlations between PAF Enzymes at Baseline and at Week 8

At baseline a positive correlation was observed between PAF-CPT and PAF-AH (*r* = 0.287, *p* = 0.008), and a borderline positive correlation was found between Lyso-PAF AT in the presence of EDTA and PAF-AH (*r* = 0.210, *p* = 0.054), after adjustments for sex, age and BMI. At week 8 of the study Lyso-PAF AT isoform activated in the presence of Ca^2+^ showed a negative correlation with PAF-CPT (*r* = −0.552, *p* = 0.003) and PAF-AH (*r* = −0.385, *p* = 0.048) in the enriched yogurt group.

## 4. Discussion

Nowadays, early identification of individuals at risk of developing CVDs, and implementation of effective treatments is the prime objective of medical research [1]. Consumption of dairy products has been associated with neutral or beneficial effects against CVD risk [41,42]. However, there is a lack of sufficient evidence with respect to the specific bioactive compounds [29], and the biological pathways involved in CVDs risk reduction, as well as the specific types of dairy products in relation to CVDs [41].

The present study suggests that a relatively short dietary intervention with a yogurt enriched with olive pomace origin PAF inhibitors has a modest effect on PAF metabolism by lowering the activity of platelet-activating factor–cholinephosphotransferase as well as plasma LpPLA_2_ activity.

The activities of PAF metabolic enzymes are comparable to the ones reported for apparently healthy Greek population with similar age and BMI values [37] with the exception of Lyso-PAF AT where no direct comparison can be made since the isoforms of Lyso-PAF AT were not evaluated in the study of Detopoulou et al. In the present study, Lyso-PAF ATE activity is in the same range reported from Gavriil et al. [25] while Lyso-PAF ATC activity is much higher in our participants probably due to the fact that they are older and with higher BMI values, even though no relationship between either age or BMI was detected. The activity of LpPLA_2_ is lower than that reported for Greek subjects in the ATTICA study [43], but it is similar with other studies [25,37,44]. In the present work, LpPLA_2_ activity was lower in women than men, and this is in accordance with all previous studies [37,43,44]. In addition, we should point out that dietary macronutrient intake and physical activity remained similar and unaffected during all intervention groups (data not shown) since it has been reported that total energy intake is a potential determinant of LpPLA_2_ activity [44].

A growing body of evidence indicates that LpPLA_2_ represents an independent risk factor for CVDs [11,45]. Circulating LpPLA_2_ is primarily associated with LDL-C with the majority bound to atherogenic small dense LDL particles [46]. The LpPLA_2_ activity taking under consideration the LDL levels (LpPLA_2_-to-LDL ratio) was lower after consumption of the yogurt enriched with PAF inhibitors from olive pomace compared to the plain yogurt at the end of the intervention. This finding is in accordance with other studies where dietary interventions resulted in reduced LpPLA_2_ levels/activity [47,48,49]. In volunteers with metabolic syndrome red yeast rice olive supplementation is reported to inhibit in vivo LpPLA_2_ activity [50]. Nelson et al. showed that supplementation with ω-3 fatty acid capsules over 8 weeks had no significant effect on plasma LpPLA_2_ activity when compared to the control capsule that included olive oil [51]. 

The intake of the enriched yogurt also resulted in reduced PAF-CPT activity both in the middle and at the end of the intervention compared to the control group while the intake of plain yogurt presents a modest effect on PAF-CPT only at the end of the intervention. This outcome is in accordance with the study of Tsoupras et al. [52], where incubation of human mesangial cells with the same polar lipid extract from olive pomace, resulted in significant decrease of PAF-CPT activity. In addition, hypercholesterolemic rabbits fed with the enriched yogurt used in the present study, presented lower activity of PAF-CPT and PAF-AH in leukocytes as well as reduced atheromatic lesions thickness [53]. Similar results with respect to PAF-CPT and Lyso-PAF AT specific activity were obtained in six healthy non-smoker men who received 20 mg/day simvastatin orally for 3 weeks. Specifically, simvastatin administration caused a 72% reduction of the specific activity of PAF-CPT in human leukocyte homogenates, whereas the specific activity of Lyso-PAF AT in human leucocyte homogenates did not change [54]. Based on these findings, the authors suggested that bioactive compounds with anti-PAF activity may suppress the de novo pathway of PAF biosynthesis, whereas they do not affect the remodeling pathway among healthy subjects [54]. However, in a clinical trial among young, non-obese healthy men, wine consumption, along with a high fat meal (53% of the total energy intake), decreased the activity of both PAF biosynthetic enzymes in the postprandial state compared to the ethanol solution trial [24]. In the previous studies, it was also reported that the two main PAF biosynthetic enzymes namely PAF-CPT and PAF-AT are positively correlated with each other mainly in men [22,25,52] and a positive correlation between PAF levels and PAF-CPT activity was detected [52]. In our study, a positive correlation was observed between PAF-CPT and PAF-AH, and a borderline positive correlation was found between Lyso-PAF AT in the presence of EDTA and PAF-AH, adjusted for sex, age and BMI before the intervention, suggesting the existence of a balancing system at intracellular levels of PAF in leukocytes under basal conditions. At the end of the intervention, Lyso-PAF AT isoform activated in the presence of Ca^2+^ showed a negative correlation with PAF-CPT and PAF-AH in the enriched yogurt group, indicating more complex interactions between intake of PAF-inhibitors and its metabolism under inflammatory conditions. Since no data exist on the effect of even plain yogurt on PAF levels and metabolism, more studies should be conducted to elucidate the possible mechanisms.

## 5. Limitations and Strengths

Participants were mostly middle-aged, of Greek ethnicity, without history of CVDs; caution is thus needed in the extrapolation of the findings to different populations. Thus, our results are difficult to generalize in patients or in young or elderly healthy subjects. Compliance of participants to the study protocol was estimated by phone calls and not by methods that measured specific nutrients. We did not measure levels of PAF or oxidized phospholipids. Nevertheless, this study has also strengths, such as the study design. Any potential sources of bias (i.e., selection, performance and detection bias), were eliminated due to the design of the study (i.e., randomized, double-blind). Moreover, attrition bias (i.e., systematic differences between groups in withdrawals) was also not evident here, whereas, ITT analysis was followed-on, and all results were reported (diluting reporting bias). Another strength of this study is that the activity of both isoforms of Lyso-PAF AT, the crucial enzyme in the remodeling pathway of PAF biosynthesis, was examined. Additionally, to our knowledge, this is the first study that investigated the impact of a novel enriched yogurt in PAF metabolism in an appreciable number of volunteers.

## 6. Conclusions

In conclusion, this is the first study to evaluate PAF metabolic enzymes (leukocyte PAF-CPT, Lyso-PAF ATs, PAF-AH and serum LpPLA_2_) in overweight healthy volunteers after daily consumption of yogurt. Intake of an enriched yogurt with PAF inhibitors from plant origin reduced PAF’s biosynthetic enzyme (PAF-CPT) and PAF’s catabolic enzyme (LpPLA_2_). These changes possibly reflect lower PAF levels and suggest a favorable anti-thrombotic and cardioprotective role of PAF inhibitors.

## Figures and Tables

**Table 1:** Baseline characteristics of participants randomly assigned to each intervention (ITT analysis).

Variable	Control (*n* = 30)	Plain Yogurt (*n* = 28)	Enriched Yogurt (*n* = 30)	*p*_between-group_
Men (*n*)	16	14	16	0.958
Age (years)	49.5 (8.8)	48.8 (8.9)	46.8 (8.3)	0.463
BMI (kg/m^2^)	27.2 (23.5, 30.2)	29.4 (25.5, 31.3)	26.8 (24.9, 29.5)	0.316
LDL-C (mg/dL)	134.4 (35.6)	133.2 (25.5)	140.4 (34.0)	0.657
PAF-AH (pmol/mg/min)	63.68 (45.55, 80.08)	51.45 (36.70, 76.86)	66.05 (36.28, 80.90)	0.613
LpPLA_2_ (nmol/mL/min)	26.46 (22.20, 29.37)	27.80 (23.65, 30.90)	27.69 (23.77, 29.15)	0.330
PAF-CPT (pmol/mg/min)	179.23 (135.31, 207.86)	154.08 (115.89, 251.77)	154.34 (94.0, 189.0)	0.470
Lyso-PAF ATC (pmol/mg/min)	131.15 (64.48, 182.13)	98.58 (84.48, 113.54)	76.75 (60.83, 104.13)	0.012
Lyso-PAF ATE (pmol/mg/min)	41.45 (30.26, 86.08)	56.10 (33.39, 79.67)	51.75 (33.78, 83.29)	0.919
LpPLA_2_-to-LDL ratio	0.18 (0.15, 0.22)	0.21 (0.19, 0.25)	0.19 (0.18, 0.23)	0.095

Categorical variables are presented as absolute frequencies (*n*); continuous variables are presented as mean (standard deviation) if normally distributed or as median (25th, 75th percentiles) if non-normally distributed; Pearson’s chi-square test was used to assess the association between sex and intervention group; one-way analysis of variance (ANOVA) or Kruskal–Wallis tests were used to compare groups for continuous variables with normal and non-normal distribution, respectively; significance (α) level was set to 0.05. BMI: body mass index; LDL-C: low-density lipoprotein cholesterol; LpPLA_2_: lipoprotein-associated phospholipase A_2_; Lyso-PAF ATC: lyso-platelet-activating factor acetyltransferase in the presence of Ca^2+^; Lyso-PAF ATE: lyso-platelet-activating factor acetyltransferase in the presence of EDTA; PAF-AH: platelet-activating acetyl-hydrolase; PAF-CPT: platelet-activating factor-cholinephosphotransferase.

**Table 2:** Characteristics of the participants during the 4th week of the dietary intervention (ITT analysis).

Variable	Control (*n* = 30)	Plain Yogurt (*n* = 28)	Enriched Yogurt (*n* = 30)	*p*_between-group_
BMI (kg/m^2^)	27.1 (23.7, 30.4)	29.4 (25.5, 31.2)	27.0 (24.8, 29.7)	0.333
LDL-C (mg/dL)	130.8 (38.6)	132.1 (26.5)	135.7 (32.9)	0.838
PAF-AH (pmol/mg/min)	79.05 (56.68, 93.58)	48.20 (39.74, 78.76)	60.97 (41.35, 86.27)	0.069
LpPLA_2_ (nmol/mL/min)	27.24 (19.06, 31.17)	27.47 (24.66, 30.60)	26.68 (23.32, 29.37)	0.684
PAF-CPT (pmol/mg/min)	173.65 (138.23, 219.46)	142.44 (112.61, 203.06)	155.22 (90.85, 175.02)	0.132
Lyso-PAF ATC (pmol/mg/min)	122.31 (79.69, 169.50)	85.51 (65.07, 110.82)	79.10 (51.61, 121.41)	0.036
Lyso-PAF ATE (pmol/mg/min)	44.36 (33.57, 78.48)	56.83 (36.12, 68.89)	46.65 (36.77, 73.30)	0.999
LpPLA_2_-to-LDL ratio	0.19 (0.15, 0.25)	0.21 (0.19, 0.24)	0.20 (0.17, 0.22)	0.232

Categorical variables are presented as absolute frequencies (*n*); continuous variables are presented as mean (standard deviation) if normally distributed or as median (25th, 75th percentiles) if non-normally distributed; one-way analysis of variance (ANOVA) or Kruskal–Wallis tests were used to compare groups for continuous variables with normal and non-normal distribution, respectively; significance (α) level was set to 0.05. BMI: body mass index; LDL-C: low-density lipoprotein cholesterol; LpPLA_2_: lipoprotein-associated phospholipase A_2_; Lyso-PAF ATC: lyso-platelet-activating factor acetyltransferase in the presence of Ca^2+^; Lyso-PAF ATE: lyso-platelet-activating factor acetyltransferase in the presence of EDTA; PAF-AH: platelet-activating acetyl-hydrolase PAF-CPT: platelet-activating factor-cholinephosphotransferase.

**Table 3:** Characteristics of the participants during the 8th week of the dietary intervention (ITT analysis).

Variable	Control (*n* = 30)	Plain Yogurt (*n* = 28)	Enriched Yogurt (*n* = 30)	*p*_between-group_
BMI (kg/m^2^)	26.9 (23.4, 30.2)	29.0 (25.4, 31.1)	27.2 (24.8, 29.5)	0.338
LDL-C (mg/dL)	130.3 (38.0)	131.4 (27.8)	136.5 (34.9)	0.751
PAF-AH (pmol/mg/min)	67.38 (46.07, 86.46)	50.13 (34.87, 74.81)	55.0 (35.72, 75.30)	0.148
LpPLA_2_ (nmol/mL/min)	27.13 (23.54, 31.84)	29.15 (24.44, 32.41)	26.23 (21.97, 30.49)	0.300
PAF-CPT (pmol/mg/min)	183.87 (144.25, 227.20)	151.81 (104.18, 201.85)	146.44 (130.23, 190.51)	0.094
Lyso-PAF ATC (pmol/mg/min)	117.27 (64.92, 176.40)	87.76 (68.77, 126.33)	78.60 (55.73, 108.04)	0.106
Lyso-PAF ATE (pmol/mg/min)	44.13 (34.10, 74.10)	56.86 (41.45, 83.58)	55.21 (38.85, 70.83)	0.601
LpPLA_2_-to-LDL ratio	0.20 (0.17, 0.25)	0.23 (0.20, 0.26)	0.19 (0.17, 0.22)	0.049

Categorical variables are presented as absolute frequencies (*n*); continuous variables are presented as mean (standard deviation) if normally distributed or as median (25th, 75th percentiles) if non-normally distributed; one-way analysis of variance (ANOVA) or Kruskal–Wallis tests were used to compare groups for continuous variables with normal and non-normal distribution, respectively; significance (α) level was set to 0.05. BMI: body mass index; LDL-C: low-density lipoprotein cholesterol; LpPLA_2_: lipoprotein-associated phospholipase A_2_; Lyso-PAF ATC: lyso-platelet-activating factor acetyltransferase in the presence of Ca^2+^; Lyso-PAF ATE: lyso-platelet-activating factor acetyltransferase in the presence of EDTA; PAF-AH: platelet-activating acetyl-hydrolase; PAF-CPT: platelet-activating factor-cholinephosphotransferase.

**Table 4:** Estimation of the difference in the enzyme activities between the intervention groups evaluated at week 4 of the study.

Variable	Enriched Yogurt vs. Control	Plain Yogurt vs. Control	Enriched vs. Plain Yogurt
PAF-AH (pmol/mg/min)	−3.90 (−8.47, 0.66)	−9.88 (−23.05, 3.28)	2.59 (−10.83, 16.01)
LpPLA_2_ (nmol/mL/min)	−0.43 (−1.14, 0.28)	−0.28 (−1.82, 1.25)	−0.65 (−2.48, 1.18)
PAF-CPT (pmol/mg/min)	−6.48 (−12.04, −0.92)	−16.36 (−33.82, 1.11)	5.14 (−6.91, 17.19)
Lyso-PAF ATC (pmol/mg/min)	−0.96 (−13.38, 11.45)	−11.81 (−30.11, 6.49)	−11.16 (−31.07, 8.75)
Lyso-PAF ATE (pmol/mg/min)	−0.22 (−3.23, 2.78)	−2.28 (−8.92, 4.36)	3.24 (−4.82, 11.30)
LpPLA_2_-to-LDL ratio	−0.003 (−0.009, 0.004)	−0.001 (−0.015, 0.013)	−0.005 (−0.012, 0.003)

Median regression was performed for each pair of intervention groups; adjustments were made for sex and enzyme variable values at baseline. Results are presented as difference in medians (95% confidence interval of the difference) between intervention groups. Significance (α) level was set to 0.05. BMI: body mass index; LDL: low-density lipoprotein; LpPLA_2_: lipoprotein-associated phospholipase A_2_; Lyso-PAF ATC: lyso-platelet-activating factor acetyltransferase in the presence of Ca^2+^; Lyso-PAF ATE: lyso-platelet-activating factor acetyltransferase in the presence of EDTA; PAF-AH: platelet-activating acetyl-hydrolase; PAF-CPT: platelet-activating factor-cholinephosphotransferase.

**Table 5:** Estimation of the difference in the enzyme activities between the intervention groups evaluated at week 8 of the study.

Variable	Enriched Yogurt vs. Control	Plain Yogurt vs. Control	Enriched vs. Plain Yogurt
PAF-AH (pmol/mg/min)	−4.88 (−11.30, 1.54)	−7.48 (−20.80, 5.85)	−1.89 (−12.30, 8.53)
LpPLA_2_ (nmol/mL/min)	−0.93 (−1.90, 0.03)	−0.72 (−2.46, 1.02)	−1.45 (−3.69, 0.79)
PAF-CPT (pmol/mg/min)	−10.58 (−20.81, −0.35)	−19.15 (−38.15, −0.14)	5.0 (−10.97, 20.97)
Lyso-PAF ATC (pmol/mg/min)	−2.68 (−11.83, 6.48)	−9.19 (−34.47, 16.10)	−3.48 (−23.19, 16.24)
Lyso-PAF ATE (pmol/mg/min)	1.93 (−0.55, 4.42)	0.93 (−5.05, 6.91)	−0.02 (−6.43, 6.39)
LpPLA_2_-to-LDL ratio	−0.005 (−0.015, 0.005)	0.0001 (−0.017, 0.017)	−0.019 (−0.034, −0.003)

Median regression was performed for each pair of intervention groups; adjustments were made for sex and enzyme variable values at baseline. Results are presented as difference in medians (95% confidence interval of the difference) between intervention groups. Significance (α) level was set to 0.05. BMI: body mass index; LDL: low-density lipoprotein; LpPLA_2_: lipoprotein-associated phospholipase A_2_; Lyso-PAF ATC: lyso-platelet-activating factor acetyltransferase in the presence of Ca^2+^; Lyso-PAF ATE: lyso-platelet-activating factor acetyltransferase in the presence of EDTA; PAF-AH: platelet-activating acetyl-hydrolase; PAF-CPT: platelet-activating factor-cholinephosphotransferase.

## Data Availability

The data presented in this study are available on request from the corresponding author. The data are not publicly available in accordance with consent provided by participants on the use of confidential data.

## Supplementary Materials

The following are available online at https://www.mdpi.com/article/10.3390/biom11060801/s1, Figure S1: CONSORT flow diagram; Figure S2: Distribution of each outcome variable measured at baseline (i.e., specific activity of PAF enzymes, and LpPLA_2_-to-LDL ratio) in women (*n* = 42) and men (*n* = 46); Figure S3: Correlations between the outcome variables measured at baseline (i.e., specific activity of PAF enzymes, and LpPLA_2_-to-LDL ratio), and age (in years) of participants (*n* = 88); Figure S4: Correlations between the outcome variables measured at baseline (i.e., specific activity of PAF enzymes, and LpPLA_2_-to-LDL ratio), and baseline BMI (in kg/m^2^) of participants (*n* = 88); CONSORT 2010 checklist of information to include when reporting a randomized trial.
